# Supplementary material for: Characterization of Blood Group Variants in an Omani Population by Comparison of Whole Genome Sequencing and Serology
Source: bioRxiv. 2024 Jun 18:2024.06.17.599396. Preprint. [Version 1] doi: 10.1101/2024.06.17.599396 (PMC11212902; doi:10.1101/2024.06.17.599396)
Supplement: Supplement 1 [file media-1.pdf]

Supplemental Tables

Table S1. Graded serology (0-4) for ABO, RHD, RHCE, Kell, Kidd, Duffy, Lewis, Lutheran, MNS, and P1, blood group systems for all 100 Omani blood donors.

Research sequence#	Blood group	Rh+K				P	Lewis		Lutheran		Kell				Kid		MNS				Duffy	
		C	c	E	e	P1	Le(a)	Le(b)	Lu(a)	Lu(b)	K	k	Kp(a)	Kp(b)	Jk(a)	Jk(b)	M	N	S	s	Fy(a)	Fy(b)
18263X1	O Rh+	0	4	4	4	0	0	4	0	3	4	3	0	3	0	4	4	4	3	3	0	0
18263X2	O Rh+	4	4	4	4	4	4	0	0	3	0	3	0	3	0	4	4	4	3	3	0	0
18263X3	O Rh+	0	4	4	4	0	0	4	0	3	0	3	3	3	0	4	0	4	0	3	0	0
18263X4	A Rh+	0	4	4	4	4	0	2	0	3	0	3	0	3	4	0	4	4	3	3	0	0
18263X5	A Rh+	4	4	4	4	4	0	4	0	3	0	4	0	3	4	0	4	4	3	3	0	0
18263X6	O Rh+	4	4	4	4	4	0	4	0	2	0	3	0	3	4	4	4	4	1	0	0	0
18263X7	O Rh+	4	4	0	4	4	0	4	0	2	0	3	0	3	4	0	4	0	3	3	0	0
18263X8	A Rh+	4	4	0	4	0	0	4	0	3	0	3	0	3	4	0	4	4	0	3	0	0
18263X9	A Rh+	4	4	0	4	4	3	4	0	1	0	3	0	3	4	4	4	0	0	3	0	0
18263X10	A Rh+	4	4	4	4	0	0	4	0	3	0	3	0	3	4	0	4	4	3	3	0	0
18263X11	O Rh+	4	0	0	4	4	0	0	0	1	0	3	0	3	0	4	4	0	3	0	0	3
18263X12	A Rh-	0	4	0	4	4	0	4	0	3	0	3	0	3	4	0	4	4	0	3	0	0
18263X13	O Rh+	4	4	0	4	4	0	0	0	3	0	3	0	3	4	0	4	4	0	3	0	0
18263X14	A Rh+	4	0	0	4	4	0	0	0	3	0	3	0	3	4	0	4	0	3	0	0	0
18263X15	O Rh+	4	0	0	4	0	0	4	0	3	0	3	0	3	4	4	4	0	3	0	0	0
18263X16	AB Rh+	0	4	0	4	4	0	3	0	3	0	3	0	3	0	4	4	4	3	3	3	0
18263X17	O Rh+	4	4	0	4	4	0	0	0	3	0	3	0	3	4	4	3	4	0	3	0	3
18263X18	O Rh+	4	0	0	4	3	0	0	0	3	0	3	0	3	0	4	4	0	3	0	0	0
18263X19	O Rh-	0	4	0	4	3	0	4	0	3	0	3	0	3	4	0	4	0	3	3	0	0
18263X20	O Rh+	4	0	0	4	4	4	0	0	3	0	3	0	3	0	4	4	0	3	3	0	0
18263X21	A Rh+	4	0	0	4	4	0	4	0	3	0	3	0	3	4	0	4	4	0	3	0	0
18263X22	O Rh+	4	4	4	4	4	0	4	0	3	0	3	0	3	4	4	4	4	0	3	0	0
18263X23	B Rh+	4	0	0	4	3	0	4	2	3	0	3	0	3	4	4	4	4	3	3	0	0
18263X24	A Rh+	4	4	4	4	4	0	3	0	1	0	4	0	3	4	4	4	4	0	3	0	0
18263X25	O Rh+	4	4	0	4	4	0	4	0	1	4	3	0	3	4	4	4	0	3	0	0	0
18263X26	O Rh+	0	4	0	4	4	4	0	0	2	0	3	0	4	4	0	4	4	0	3	0	0
18263X27	B Rh+	0	4	4	0	4	0	4	0	1	0	3	0	3	4	0	4	3	0	0	0	0
18263X28	O Rh+	4	4	0	4	4	0	4	0	3	0	3	0	3	4	4	4	4	3	3	0	0
18263X29	A Rh+	0	4	0	4	4	4	0	0	3	0	3	0	3	4	0	0	4	0	3	0	3
18263X30	O Rh+	4	4	0	4	4	0	4	0	3	4	3	0	3	0	4	4	0	3	3	0	3

18263X31	O Rh+	4	4	0	4	4	0	4	0	3	0	3	0	3	4	0	4	4	3	3	0	0
18263X32	O Rh+	4	0	0	4	0	3	0	0	3	0	3	0	3	4	0	4	1	3	0	0	0
18263X33	AB Rh+	0	4	0	4	4	0	3	0	2	0	3	0	3	4	0	4	0	3	3	0	0
18263X34	O Rh+	4	0	4	4	2	3	0	0	0	0	3	0	3	0	4	4	4	0	3	0	0
18263X35	O Rh+	4	0	0	4	0	0	4	0	1	0	3	0	3	4	0	4	4	3	0	0	0
18263X36	O Rh+	4	4	0	4	0	0	0	0	2	0	3	0	3	0	4	3	3	3	0	0	0
18263X37	O Rh+	4	0	0	4	3	0	4	0	2	0	3	0	3	4	3	3	3	0	3	0	0
18263X38	O Rh+	0	4	4	4	3	3	0	0	2	0	2	0	3	3	3	3	0	3	3	0	0
18263X39	O Rh+	4	4	4	4	3	0	4	0	2	0	3	0	3	4	0	2	3	3	3	0	0
18263X40	A Rh-	0	4	0	4	3	0	3	0	2	0	3	0	2	3	0	4	0	3	3	3	0
18263X41	O Rh+	4	0	0	4	0	0	0	0	2	0	3	0	3	4	0	3	0	3	3	0	0
18263X42	O Rh+	0	4	0	4	2	0	4	0	2	0	3	0	3	3	3	3	0	0	3	0	0
18263X43	A Rh+	4	0	0	4	3	0	4	0	2	0	3	0	3	4	0	3	0	2	0	0	0
18263X44	O Rh+	4	4	0	4	3	0	4	0	2	0	3	0	3	3	3	3	0	2	2	0	0
18263X45	A Rh+	4	0	0	4	3	0	3	0	2	0	3	0	3	4	0	3	0	2	2	3	2
18263X46	AB Rh+	4	0	0	4	2	0	0	0	2	0	3	0	3	3	3	3	0	0	3	0	0
18263X47	O Rh+	4	4	0	4	3	0	0	0	2	0	3	0	3	3	3	3	0	2	2	0	0
18263X48	AB Rh+	4	0	0	4	3	0	0	0	2	0	3	0	3	3	0	4	0	3	0	0	0
18263X49	O Rh+	4	4	0	4	2	0	3	0	2	0	3	0	3	4	3	3	3	2	2	0	0
18263X50	O Rh+	4	0	0	4	3	0	0	0	2	0	2	0	3	4	3	2	3	2	0	0	0
18263X51	A Rh-	0	4	0	4	3	0	3	0	2	0	3	0	3	4	0	3	0	2	0	0	0
18263X52	O Rh+	4	4	0	4	3	0	4	0	3	0	3	0	3	4	3	4	0	0	2	0	0
18263X53	O Rh-	0	4	0	4	0	2	0	0	2	0	2	0	3	4	0	4	0	3	2	0	0
18263X54	O Rh+	4	0	0	4	4	0	0	0	3	0	3	0	3	4	3	4	0	3	0	0	0
18263X55	O Rh+	4	0	0	4	0	3	0	0	2	0	3	0	3	3	4	4	0	0	3	0	0
18263X56	B Rh+	4	4	0	4	3	2	0	0	2	0	3	0	3	4	3	3	3	0	3	0	0
18263X57	O Rh+	0	4	0	4	3	0	3	0	2	4	3	0	2	4	0	3	3	3	3	0	0
18263X58	O Rh+	4	0	0	4	0	0	3	0	2	0	3	3	2	4	0	2	2	2	2	2	0

18263X59	A Rh+	4	0	0	4	2	0	3	0	2	0	3	0	3	4	0	3	0	2	3	0	0
18263X60	O Rh+	4	0	0	4	3	0	4	0	2	0	3	0	3	3	4	3	0	2	2	0	0
18263X61	B Rh+	0	4	4	4	3	3	0	0	2	0	2	0	3	3	3	4	0	2	2	0	0
18263X62	O Rh+	4	4	0	4	3	0	4	0	3	0	3	0	3	4	0	3	3	2	2	0	0
18263X63	B Rh+	4	0	0	4	4	0	3	0	3	4	2	0	3	0	3	3	0	2	2	0	0
18263X64	B Rh+	0	4	4	0	4	0	3	0	2	0	3	0	2	0	3	0	3	2	3	0	0
18263X65	O Rh-	0	4	0	4	3	3	0	0	2	0	3	0	2	4	0	2	3	3	2	0	0
18263X66	A Rh-	4	4	0	4	2	0	0	0	2	0	3	0	3	4	0	2	3	2	0	2	0
18263X67	O Rh+	0	4	4	4	4	0	4	0	2	0	3	0	3	3	3	2	3	2	2	0	0
18263X68	O Rh+	4	4	0	4	0	0	3	0	3	0	3	0	3	4	0	2	3	0	2	0	0
18263X69	O Rh-	0	4	0	4	2	0	3	0	2	0	3	0	3	4	4	3	0	0	3	0	2
18263X70	O Rh+	4	4	4	4	0	0	4	0	2	4	2	0	3	4	0	3	0	2	3	0	0
18263X71	O Rh+	4	4	0	4	3	0	0	0	2	0	3	0	3	4	0	2	3	2	2	0	0
18263X72	B Rh+	0	4	4	4	0	0	3	0	2	0	3	0	3	3	0	2	3	0	2	0	2
18263X73	A Rh+	4	0	0	4	3	0	4	0	2	0	3	0	3	4	0	3	0	2	3	2	0
18263X74	O Rh+	4	4	0	4	3	0	4	0	2	0	3	0	3	0	4	3	3	3	3	0	0
18263X75	B Rh+	0	4	4	4	0	0	4	0	2	0	3	0	2	4	4	3	3	3	3	0	0
18263X76	B Rh+	4	4	0	4	2	0	3	0	2	0	3	0	3	4	0	3	3	0	3	0	0
18263X77	O Rh+	4	4	0	4	3	0	0	0	3	0	3	0	2	3	3	2	3	2	3	0	0
18263X78	B Rh+	4	4	4	4	4	0	3	0	2	0	3	0	3	0	3	4	0	0	3	0	0
18263X79	O Rh+	4	4	0	4	3	0	4	0	2	0	3	0	3	0	4	3	3	0	3	0	0
18263X80	O Rh+	4	0	0	4	2	3	0	0	2	0	3	0	3	4	3	3	3	2	2	0	0
18263X81	O Rh+	4	0	0	4	3	0	4	0	2	0	3	0	3	4	0	3	3	3	3	2	0
18263X82	O Rh+	0	4	4	4	3	0	4	0	2	0	3	0	2	4	0	2	2	0	2	0	0
18263X83	O Rh+	0	4	0	4	4	0	4	0	2	0	3	0	3	4	4	4	0	3	0	0	0
18263X84	O Rh+	4	4	0	4	4	0	4	0	2	0	3	0	3	4	0	2	2	0	2	0	0
18263X85	A Rh+	0	4	0	4	2	0	4	0	2	0	2	0	3	4	0	3	2	0	3	0	0
18263X86	O Rh+	4	0	0	4	4	0	4	0	2	0	2	0	3	3	3	4	0	3	1	0	0

18263X87	O Rh+	4	4	0	4	0	0	4	0	2	0	3	0	3	4	0	4	2	0	3	0	0
18263X88	A Rh+	4	4	0	4	2	0	3	0	2	0	3	0	3	4	0	2	2	0	3	0	0
18263X89	O Rh+	4	4	0	4	0	0	4	0	2	0	3	0	3	4	3	4	0	2	2	0	0
18263X90	A Rh+	4	4	4	4	2	0	4	0	2	0	2	0	2	3	3	2	0	2	2	0	0
18263X91	O Rh+	4	4	0	4	3	3	0	0	4	0	3	0	3	4	3	4	0	0	3	0	0
18263X92	O Rh+	4	4	0	4	0	0	3	0	2	0	3	0	3	2	2	3	2	0	2	0	0
18263X93	B Rh+	4	0	0	4	3	0	3	0	2	4	2	0	3	3	3	2	0	0	3	0	0
18263X94	O Rh+	0	4	0	4	3	0	4	0	2	0	3	0	3	4	3	0	4	3	3	0	0
18263X95	O Rh+	0	4	0	4	3	0	4	2	2	0	3	0	3	4	0	0	3	0	3	0	0
18263X96	B Rh+	0	4	0	4	0	0	4	2	2	0	3	0	2	0	3	3	3	0	2	0	0
18263X97	B Rh+	4	4	0	4	0	0	0	0	2	0	2	0	3	4	3	0	3	0	3	0	0
18263X98	O Rh+	0	4	4	4	3	0	4	0	2	0	3	0	3	4	4	3	2	0	2	0	0
18263X99	O Rh+	0	4	0	4	3	0	0	0	2	0	2	0	3	4	3	3	2	2	0	0	0
18263X100	O Rh+	4	0	0	4	4	0	4	0	2	0	2	0	3	2	3	4	0	2	0	0	0

Table S2. Genotypes and serological phenotypes for discordant samples using commonly typed variants to infer blood group phenotypes.

Sample Count	Blood Group	Phenotype by Serology	Phenotype inferred from genotype	Genotype	Resolved?
1	RHD	D-	D+	Two Copies of *RHD*	Yes: rs748783394
1	RHCE (C/c)	C+c+	C-c+	Two Copies of RHCE Exon 2	No
1	RHCE (E/E)	E+e+	E-e+	rs609320: 0/0	Yes: rs141398055
4	Duffy	Fy(a-b-)	Fy(a-b+)	rs2814778: 0/1 rs12075: 1/1	Yes
1	Lewis	Le(a+b+)	Le(a-b+)	rs601338: 0/1 rs1047781: 0/0 rs28362459: 0/0 rs812936: 0/1 rs778986: 0/1	Candidate: rs373779096
1	Lewis	Le(a-b-)	Le(a-b+)	rs601338: 0/0 rs1047781: 0/0 rs28362459: 0/1 rs812936: 0/1 rs778986: 0/1	Yes: rs3894326
1	Lewis	Le(a-b-)	Le(a-b+)	rs601338: 0/1 rs1047781: 0/0 rs28362459: 0/1 rs812936: 0/1 rs778986: 0/1	Yes: rs3894326
1	Lewis	Le(a-b-)	Le(a+b-)	rs601338: 1/1 rs1047781: 0/0 rs28362459: 0/1 rs812936: 0/1 rs778986: 0/1	Yes: rs3894326

1	Lutheran	In(Lu)	Lu(a-b+)	rs28399653: 0/0	Candidate: rs533045163 and rs184739796
5	MNS (M/N)	M+N+	M-N+	rs7682260: 0/0 rs7687256: 0/0 rs7658293: 0/0	No
1	MNS (M/N)	M+N+	M+N-	rs7682260: 1/1 rs7687256: 1/1 rs7658293: 1/1	No
1	MNS (S/s)	S-s-	S+s-	rs7683365: 1/1	Yes: rs139511876, DEL1
1	MNS (S/s)	S+s-	S+s+	rs7683365: 0/1	Candidate: Dantu SV
1	MNS (S/s)	S+s+	S+s-	rs7683365: 1/1	No
1	P1	P2	P1	rs66781836: 0/1 rs5751348: 0/1 rs8138197: 0/1 rs2143918: 0/1	Candidate: *A4GALT* -289A>C

References

1. Singleton BK, Green CA, Avent ND, et al. The presence of an RHD pseudogene containing a 37 base pair duplication and a nonsense mutation in africans with the Rh D-negative blood group phenotype. *Blood*. Jan 1 2000;95(1):12-8.
2. Haffener PE, Al-Riyami AZ, Al-Zadjali S, et al. Adaptive admixture at ACKR1 (the Duffy locus) may have shaped Plasmodium vivax prevalence in Oman. *bioRxiv*. 2024:2024.03.06.583766. doi:10.1101/2024.03.06.583766
3. Storry JR, Reid ME, Fetics S, Huang CH. Mutations in GYPB exon 5 drive the S-s-U+(var) phenotype in persons of African descent: implications for transfusion. *Transfusion*. Dec 2003;43(12):1738-47. doi:10.1046/j.0041-1132.2003.00585.x
4. Leffler EM, Band G, Busby GBJ, et al. Resistance to malaria through structural variation of red blood cell invasion receptors. *Science*. Jun 16 2017;356(6343)doi:10.1126/science.aam6393
